# Adeno‐associated virus‐based approach for genetic modification of cardiac fibroblasts in adult rat hearts

**DOI:** 10.14814/phy2.15989

**Published:** 2024-03-27

**Authors:** Bridget Nieto, Michael W. Cypress, Bong Sook Jhun, Jin O‐Uchi

**Affiliations:** ^1^ Cardiovascular Division, Department of Medicine, Lillehei Heart Institute University of Minnesota Minneapolis Minnesota USA

**Keywords:** cardiac fibroblasts, cardiomyocytes, in vivo gene delivery, outer mitochondrial membrane, protein kinase D

## Abstract

Cardiac fibroblasts (CFs) are an attractive target for reducing pathological cardiac remodeling, and understanding the underlying mechanisms of these processes is the key to develop successful therapies for treating the pressure‐overloaded heart. CF‐specific knockout (KO) mouse lines with a Cre recombinase under the control of human TCF21 (hTCF21) promoter and/or an adeno‐associated virus serotype 9 (AAV9)‐hTCF21 system provide a powerful tool for understanding CF biology in vivo. Although a variety of rat disease models are vital for the research of cardiac fibrosis similar to mouse models, there are few rat models that employ cardiac cell‐specific conditional gene modification, which has hindered the development and translational relevance of cardiac disease models. In addition, to date, there are no reports of gene manipulation specifically in rat CFs in vivo. Here, we report a simplified CF‐specific rat transgenic model using an AAV9‐hTCF21 system that achieved a CF‐specific expression of transgene in adult rat hearts. Moreover, we successfully applied this approach to specifically manipulate mitochondrial morphology in quiescent CFs. In summary, this model will allow us to develop fast and simple rat CF‐specific transgenic models for studying cardiovascular diseases in vivo.

## 1 INTRODUCTION

The use of rodent models remains as one of the key strategies for investigating the mechanisms of human heart disease because of their strong reproductive ability, easy detection, and economic advantage (Camacho et al., 2016; Jia et al., 2020). Overexpression and deletion of genes specifically in major cell types in the heart, such as cardiomyocytes (CMs) and cardiac fibroblasts (CFs), in mice became a top tool for studying the impact of genetics on cardiac function and dysfunction in vivo. For instance, a recent advance in temporally controlled gene ablation/overexpression in adult mice provides tremendous opportunities to investigate various genes and proteins in the heart by bypassing embryonic lethality or malformations derived from constitutive gene knockout (KO; Acharya et al., 2011; Kanisicak et al., 2016; Sohal et al., 2001). Although the use of transgenic mouse lines has become popular for cardiac research, data from these genetic models must be interpreted with caution. Because of their smaller‐sized heart, higher heartbeat rate, and short lifespan, mouse cardiac function has limited applicability to human physiology. Therefore, the use of larger rodent models, such as rats and guinea pigs, is still valuable for enhancing the translational relevance of cardiac research. A variety of rat cardiac disease models (e.g., myocardial infarction, ischemia–reperfusion, and diabetic cardiomyopathy) have proven vital for cardiac research alongside mouse models (Camacho et al., 2016). However, rat models featuring cardiac cell‐specific conditional gene modification are either lacking or not widely available, which has impeded the enhancement of cardiac disease models in rodents (Zhang et al., 2021). For instance, CM‐specific gene KO models using a transgenic rat line with Cre recombinase under a CM‐specific promoter (i.e., α‐MHC‐Cre rats) have been reported by a single group (Ma et al., 2017), but their availability is still limited.

To avoid or reduce the time‐consuming breeding strategies for generating transgenic lines, an alternative system using adeno‐associated virus (AAV) has been also developed (Inagaki et al., 2006; Piras et al., 2016; Prasad et al., 2011). Injection of AAV serotype 9 (AAV9) carrying a CM‐ or CF‐specific promoter with Cre recombinase to mice with a LoxP‐flanked (floxed) gene is a rapid and simple strategy for cardiac gene inactivation in the mouse heart (Francisco et al., 2021; Werfel et al., 2014). Importantly, this technology has started to be tested for targeting rat CMs in vivo (Schlesinger‐Laufer et al., 2020), although to date there are no reports attempting to manipulate genes in rat CFs. Here, we report a simplified CF‐specific rat transgenic model using the AAV9 system, which achieved CF‐specific transgenic expression in adult rat hearts. In addition, we successfully applied this approach to specifically manipulate mitochondrial function in quiescent CFs by introducing a mitochondria‐targeted transgene. This model will allow us to develop fast and simple rat CF‐specific transgenic models of cardiovascular diseases to precisely investigate the physiological and pathological roles of CF function in vivo.

## 2 MATERIALS AND METHODS

### 2.1 Ethical approval

All animal experiments were performed in accordance with the Guidelines on Animal Experimentation of the University of Minnesota (UMN). The study protocols were approved by Institutional Animal Care and Use Committee (IACUC) at UMN. The investigation confirmed the Guidelines for the Care and Use of Laboratory Animals published by the US National Institutes of Health (NIH).

### 2.2 Antibodies, plasmids, and reagents

All antibodies, plasmids, and viruses used in this study are listed in Tables S1–S3, respectively. All chemicals and reagents were purchased from Sigma‐Aldrich (St. Louis, MO, USA), otherwise listed in Table S4.

### 2.3 In vivo gene delivery

Adult male Sprague–Dawley rats (125–175 g; Charles River Laboratories, Wilmington, MA, USA) were anesthetized with 1%–2% isoflurane and injected with AAV9‐containing solution (1.5 × 10^13^ viral genomes/kg) via the lateral tail vein using a 28‐gauge needle (Piras et al., 2016; Prasad et al., 2011).

### 2.4 Echocardiography

Transthoracic echocardiography was performed using a Vevo 2100 and F2 ultrasound system equipped with an MS250 transducer (FUJIFILM VisualSonics, Inc., Toronto, Canada; Vang et al., 2021). All the data were analyzed with Vevo LAB software (VisualSonics; Vang et al., 2021).

### 2.5 In vivo bioluminescence imaging

Rats were anesthetized with 2% isoflurane and the images were captured with IVIS 100 In Vivo Imaging System (PerkinElmer, Waltham, MA) from 5 to 35 min after injection of 150 mg/kg D‐luciferin potassium salt (Piras et al., 2016; Prasad et al., 2011). All the animals were imaged every 5 min with the same exposure time and bioluminescence signals were quantified using the Living Image Software (PerkinElmer).

#### 2.5.1 Euthanasia

We used methods recommended by the Panel on Euthanasia of the American Veterinary Medical Association and approved by the UMN IACUC. Rats were anesthetized by the intraperitoneal injection of pentobarbital sodium and phenytoin sodium (Euthazol®, 90 mg/kg), followed by the removal of the heart.

### 2.6 Reverse transcription and quantitative PCR (RT‐qPCR)

Total RNA was extracted from rat tissues using an RNeasy Fibrous Tissue Mini Kit. Equal amounts of RNA from each sample were reverse‐transcribed to cDNA and qPCR was performed in an ABI 7500 Fast and QuantStudio 3 (Applied Biosystems, Foster City, CA) with GFP primers (F: 5′‐AAGCTGACCCTGAAGTTCATCTGC‐3′, and R: 5′‐CTTGTAGTTGCCGTCGTCCTTGAA‐3′). Cycle threshold (C_t_) values were calculated and the data for each gene were normalized against 18S expression levels (F: 5′‐GTAACCCGTTGAACCCCATT‐3′, and R: 5′‐CCATCCAATCGGTAGTAGCG‐3′).

### 2.7 Cell culture, transfection, and infection

HEK293T and H9c2 cells were maintained in Dulbecco's modified Eagle's medium supplemented with 4.5 g/L glucose, 1 mM sodium pyruvate, 1% L‐glutamine, 10% fetal bovine serum, 100 U/mL penicillin, and 100 μg/mL streptomycin at 37°C with 5% CO_2_ in a humidified incubator. For maintenance of stable cell lines, the growth medium containing the selection antibiotic 1.6 mg/mL of G418 was refreshed every 48 hrs (O‐Uchi et al., 2013). Four to 5 × 10^5^ cells plated in a 3.5‐cm dish were transfected with 3 μg of plasmids using 7 μL of FuGENE HD transfection reagent and 100 μL Opti‐MEM. Human adult ventricular cardiac fibroblasts (HCFs) from healthy donors were maintained in HCF Growth Medium at 37°C with 5% CO_2_ in a humidified incubator (Rizvi et al., 2016). One to 1.2 × 10^6^ HCFs plated in a 3.5‐cm dish were infected with adenovirus (see Table S3) at a multiplicity of infection (MOI) of 100 in the culture medium. After 3 h, the adenovirus‐infected medium was removed and replaced with fresh culture medium and used for experiments 48 h after infection.

### 2.8 Western blot, immunoprecipitation, and protein fractionation

Whole‐cell and tissue lysates were subjected to SDS‐PAGE and transferred to nitrocellulose membranes (0.45 or 0.22 μm pore size) for immunoblot analysis. Membranes were blocked with blocking buffer and treated with primary antibodies, followed by incubation with fluorescence‐conjugated secondary antibodies. Target protein bands were visualized, and the digital data were obtained using an Odyssey infrared imaging system (LI‐COR Biotechnology, Lincoln, NE). Quantitative densitometry was performed using Fiji (Schindelin et al., 2012). Immunoprecipitation (IP) of GFP from whole‐tissue lysates (500 μg) was performed with anti‐GFP antibody and protein A/G Plus agarose beads (Jhun et al., 2018). Mitochondria‐enriched and cytosolic fractions of HEK293T cells were obtained by protein fractionation using differential centrifugation (Jhun et al., 2018; O‐Uchi et al., 2013). Uncropped images of all western blotting data as Figure S1.

### 2.9 Live cell imaging and quantification of mitochondrial morphology

HCFs were stained with Mitotracker Red and observed under a laser scanning confocal microscope FV3000 with a 60× oil objective lens (Olympus, Tokyo, Japan) at room temperature with modified Tyrode's solution containing 2 mM Ca^2+^ (Jhun et al., 2018; O‐Uchi et al., 2013). Individual mitochondria from the confocal images were subjected to particle analysis to acquire the values of circularity and aspect ratio (AR: major axis/minor axis) as previously described (Jhun et al., 2018; O‐Uchi et al., 2013). The inverse of circularity was calculated to obtain the form factor (FF). Increased AR values indicate elongated tubular mitochondria and increased FF values represent increased mitochondrial branching and length. A value of 1 for both FF and AR indicates a perfect circle.

### 2.10 Immunohistochemistry

Immunostaining of the fixed ventricular tissues was prepared as we previously reported (O‐Uchi et al., 2008) and was visualized with a laser scanning confocal microscope FV3000 with 60× oil objective lens (Olympus). Control experiments were performed using secondary antibodies without primary antibodies, which showed no noticeable labeling.

### 2.11 Transmission electron microscopy (TEM)

Mitochondrial morphology in CFs in rat ventricular tissues fixed in 2% glutaraldehyde in 0.1 M phosphate buffer was assessed by TEM as we previously reported (Morimoto et al., 2009). Specimens were viewed with a Tecnai Spirit Bio‐Twin Transmission Electron Microscope (FEI Technologies Inc., Hillsboro, OR USA). Mitochondrial size and shape were assessed with Fiji software (Schindelin et al., 2012).

### 2.12 Statistics

All data in the figures are shown as the mean ± standard deviation (SD). Unpaired Student's *t*‐test was performed, and statistical significance was defined as a *p*‐value < 0.05.

## 3 RESULTS AND DISCUSSION

**FIGURE 1:**
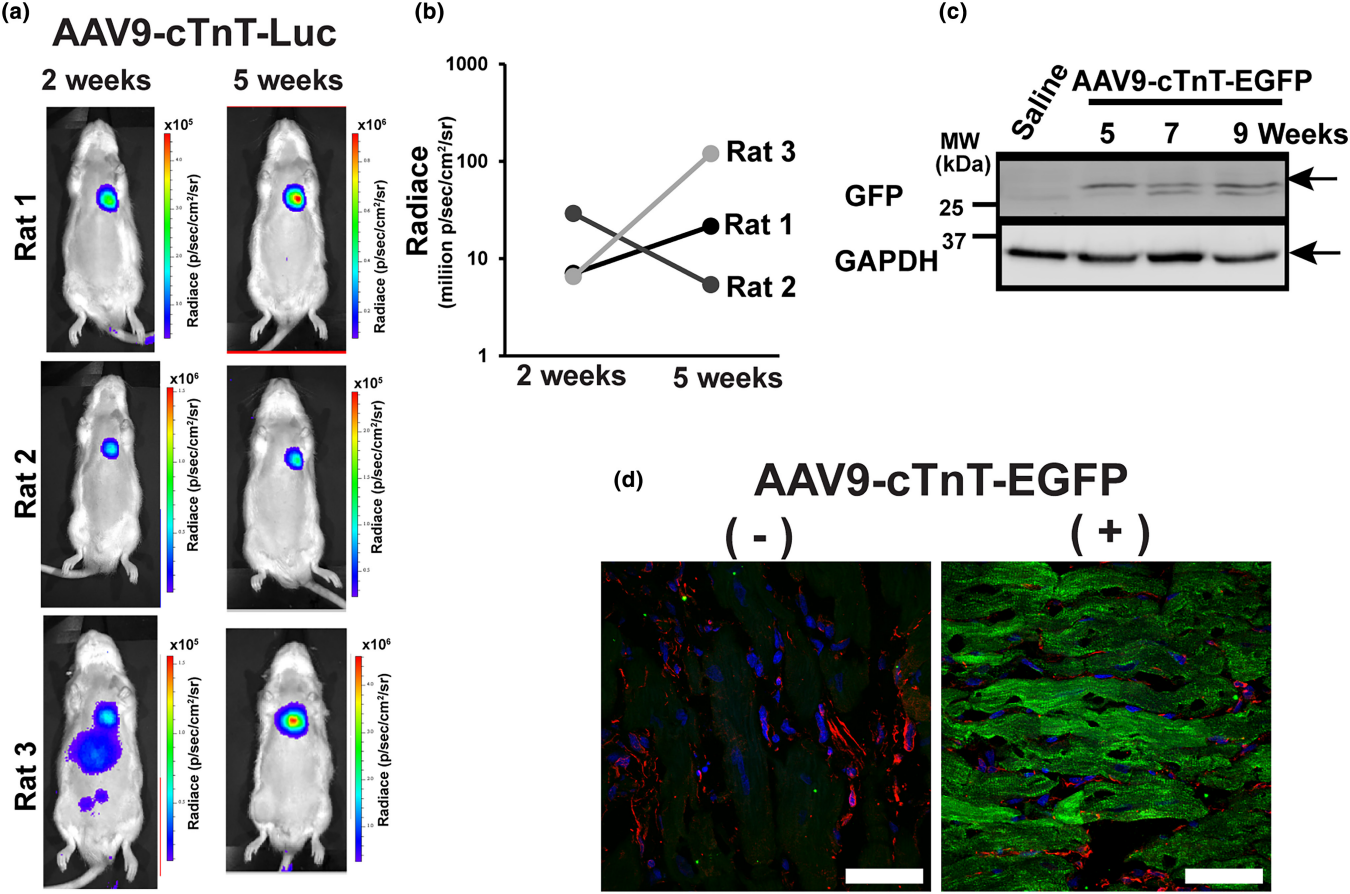
AAV9‐cTnT‐mediated in vivo gene transduction. (a) Bioluminescence images demonstrating the heart‐specific distribution of AAV9‐cTnT‐luciferase in adult rats. (b) Time course of luciferase activity at the heart area in each rat. (c) GFP expression detected by anti‐GFP antibody in heart tissue lysates. MW, molecular weight. (d) GFP expression detected by anti‐GFP antibody in ventricular sections from rats 9 weeks after the tail vein injection of AAV9‐TnT‐GFP (right) or vehicle (left). GFP, green; vimentin, red; and DAPI, blue. Representative confocal Z‐projection image from three hearts. Scale bars, 50 μm.

AAV9‐mediated gene delivery into adult mouse hearts reaches nearly maximum transduction levels in around 10 days, followed by a 50% decline over 16 weeks (Werfel et al., 2014). Based on this information, we first tested AAV9‐mediated organ‐specific (i.e., heart‐specific) in vivo gene introduction in adult rats using AAV9 carrying a CM‐specific troponin T (cTnT) promoter and luciferase (AAV9‐cTNT‐Luc) in a time‐dependent manner. In vivo noninvasive bioluminescence imaging of all three rats injected with AAV9‐cTNT‐Luc successfully exhibited luciferase activity in the hearts 2 weeks after the AAV9 injection (Figure 1a). Five weeks after injection, two out of three rats showed increased luciferase activity compared to those observed in 2 weeks (Figure 1b), indicating that the time course in AAV9‐mediated gene expression in rat hearts is more sustained compared to those in mice. One rat showed luciferase activity in the liver area in addition to the heart 2 weeks after AAV9 injection. However, this nonspecific liver luciferase activity completely disappeared in 5 weeks after AAV9 injection (Figure 1a). GFP was detectable in whole‐tissue lysates 5–9 weeks after the injection of AAV9‐cTNT‐EGFP by immunoblotting (Figure 1c). GFP was expressed in the CM area, but not in CFs (Schlesinger‐Laufer et al., 2020) in the heart tissue sections (Figure 1d), which was similar to a previous report using neonatal rats injected with AAV9‐cTNT‐EGFP (Schlesinger‐Laufer et al., 2020).

**FIGURE 2:**
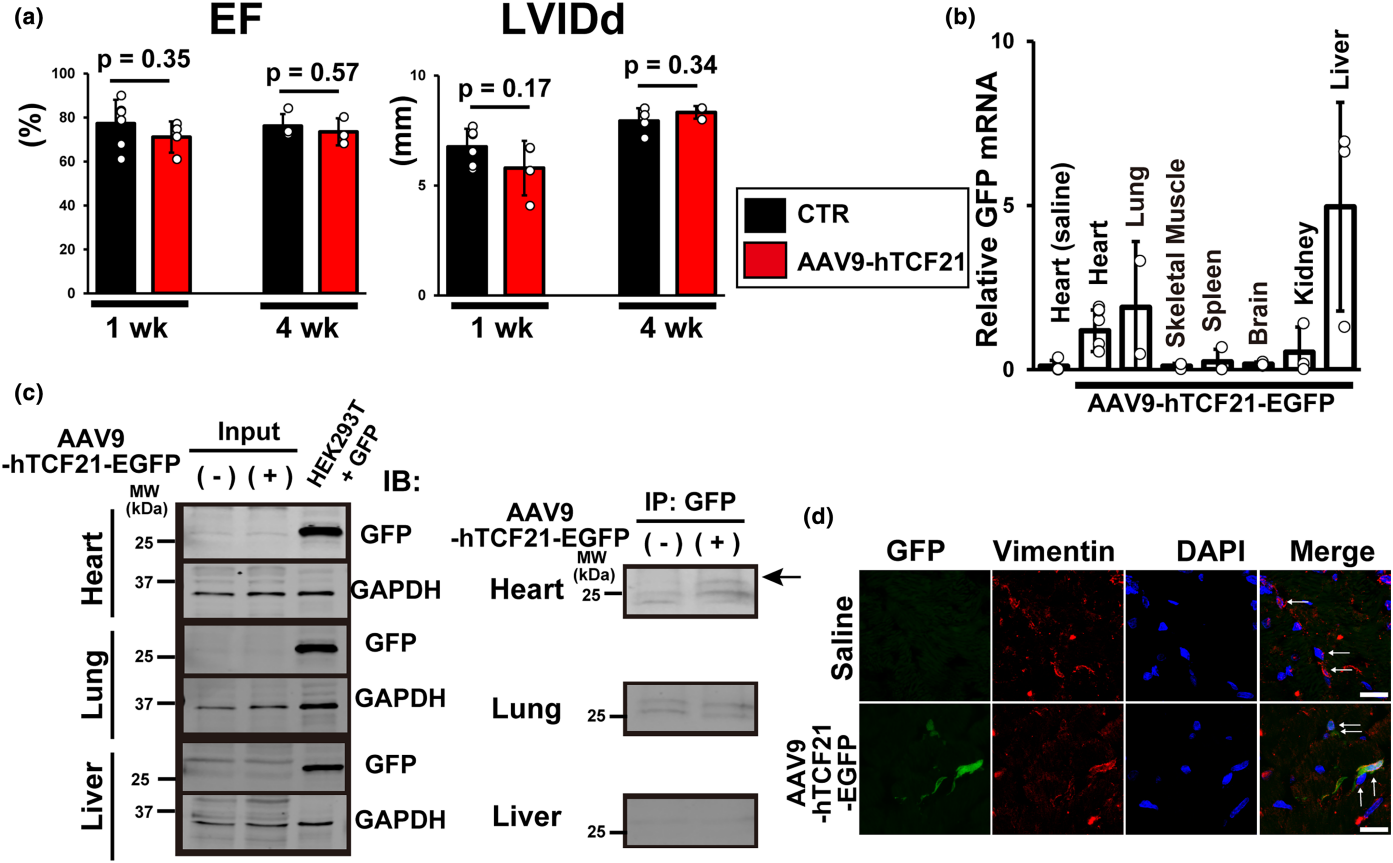
AAV9‐hTCF21‐mediated in vivo gene transduction. (a) Rats injected with AAV9‐hTCF21 have normal cardiac dimensions and function (*n* = 3–6 for each group). LVIDd, left ventricular internal diameter end diastole; EF, ejection fraction. (b) Expression of GFP mRNA in rat organs 6 weeks after tail vein injection of AAV9‐hTCF21‐GFP or saline (as a control) assessed by RT‐qPCR (*n* = 3–8). Expression level was normalized to heart with AAV9‐hTCF21‐GFP. (c) Detection of GFP protein in rat organs 6 weeks after tail vein injection of AAV9‐hTCF21‐GFP by IP. Arrows indicate the specific bands that do not exist in whole‐heart tissue lysates from saline‐injected rats. MW, molecular weight. (d) Representative confocal images of GFP in the rat ventricular tissues. The locations of CFs (arrows) were determined by vimentin (red) staining as well as nucleus staining by DAPI (blue). Representative confocal Z‐projection image from three hearts. Scale bars, 10 μm.

We next tested CF‐specific in vivo gene introduction in adult rats by utilizing the recently reported CF‐selective AAV9 carrying human TCF21 (hTCF21) promoter (Francisco et al., 2021). The echocardiographic analysis showed that AAV9‐hTCF2‐injected rats maintained normal cardiac dimensions and contractile function compared to age‐matched control rats (Figure 2a). We next investigated its organ‐specificity (i.e., heart) and cell type‐specificity (i.e., CF‐specific) in adult rats using AAV9‐hTCF21‐EGFP as organ specificity of AAV9‐hTCF21 was not documented in the original report in mice (Francisco et al., 2021). We found significant expression of GFP mRNA in the heart tissues 6 weeks after injection, but also found substantial amounts of GFP mRNA in lungs and livers, where AAV9 is reported to preferentially infect (Dayton et al., 2012; Inagaki et al., 2006; Figure 2b). The GFP protein expression was not detectable in whole‐tissue lysates of those organs (Figure 2c), but only heart lysates from AAV9‐hTCF21‐EGFP‐injected rats exhibited specific, (but faint bands) after IP with anti‐GFP antibody (Figure 2c). These results indicate that there may be off‐target cells in lungs and livers that have TCF21 activity, although the GFP expression levels are lower than in CFs. It has been shown that the TCF21 lineage develops into lipofibroblasts and interstitial fibroblasts in lungs (Park et al., 2019) and TCF21 is identified as a unique marker for quiescent hepatic satellite cells in the liver (Wang et al., 2021), which are likely the candidates that expressed GFP mRNA after AAV9‐hTCF21‐EGFP injection. Immunostaining of the ventricular tissue sections showed that GFP was only detectable in CFs (Figure 2d), but not in CM area, which was different from AAV9‐cTnT‐EGFP.

**FIGURE 3:**
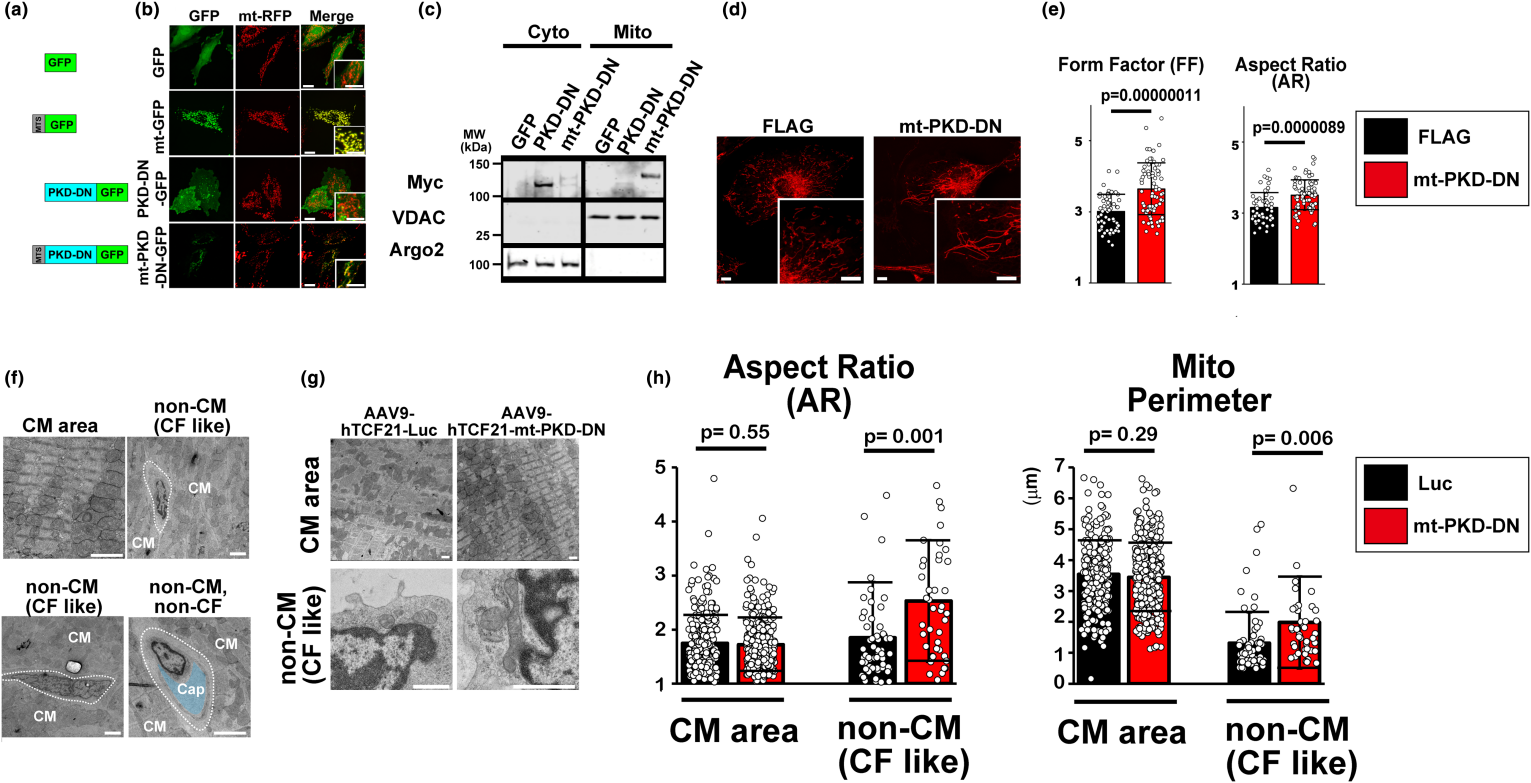
AAV9–hTCF21‐mediated in vivo gene transduction of mitochondria‐targeted dominant‐negative PKD elongates mitochondria in CFs. (a) Design of GFP‐tagged constructs. MTS, mitochondria‐targeted sequence. (b) Subcellular localization of mt‐PKD‐DN‐GFP in H9c2 cells stably overexpressing mitochondrial matrix‐targeted DsRed (mt‐RFP). GFP, mitochondrial matrix‐targeted GFP (mt‐GFP), and non‐targeted PKD‐DN‐GFP were shown as comparisons. Scale bars, 20 μm. (c) Subcellular localization of Myc‐tagged mt‐PKD‐DN and non‐tagged PKD‐DN assessed by protein fractionation. VDAC and Argonaute 2 (Argo2) were used as the markers for mitochondrial and cytosolic fractions, respectively. Note that mt‐PKD is slightly larger than non‐tagged PKD‐DN. MW, molecular weight. (d) Representative images of live HCFs stained with Mitotracker Red. Scale bars = 10 μm. (e) Summary data of FF and AR calculated from live cell imaging (*n* = 58 and 82 for FLAG [control] and mt‐PKD‐DN, respectively). (f) Representative TEM images of CM area and non‐CMs in rat ventricles. Cap, capillary structure. Scale bars, 2 μm. (g) Representative TEM images of CM area and non‐CMs (CF‐like cells) in rat ventricles 10 weeks after injection of AAV9‐hTCF21‐Luc or ‐mt‐PKD‐DN. Scale bars, 1 μm. (h) Summary data of AR and mitochondrial perimeter calculated from TEM images. *N* = 266, and 314 for Luc and mt‐PKD‐DN in CM area, respectively. *N* = 67, and 43 for Luc and mt‐PKD‐DN in CFs, respectively.

Using this AAV9‐hTCF21 system, we further tested the delivery of a genetic tool that can change the cellular function of CFs in vivo. We previously reported protein kinase D (PKD)‐dependent phosphorylation of a mitochondrial fission protein, dynamin‐related protein 1 (DRP1) at the outer mitochondrial membrane (OMM) promotes DRP1 association to the OMM, followed by mitochondrial fragmentation in cultured cardiac myoblasts (Jhun et al., 2018). A dominant‐negative mutant of PKD (PKD1‐DN) inhibits mitochondrial fission under G_q_ protein‐coupled receptor stimulation (Jhun et al., 2018). Therefore, we next generated an OMM‐targeted dominant‐negative mutant of PKD (PKD1‐DN), termed mt‐PKD‐DN, by adding an OMM‐targeting sequence obtained from human TOM20 (amino acid 1–33; Jhun et al., 2018) at the N‐terminus of PKD1‐DN (Figure 3a) to specifically inhibit PKD activity at the OMM. Using these constructs, we successfully established HEK293T and H9c2 cell lines stably overexpressing PKD1‐DN or mt‐PKD‐DN (Figure 3b,c). Stable overexpression of PKD1‐DN or mt‐PKD‐DN does not significantly change the rate of proliferation compared to cells stably expressing pcDNA3.1(+) or GFP, indicating that these constructs may not affect cell viability significantly. We confirmed the mitochondria‐specific localization of mt‐PKD‐DN by live‐cell imaging and immunoblots (Figure 3b,c). We next applied this construct to primary HCFs (Rizvi et al., 2016) and confirmed that mt‐PKD‐DN was able to significantly change mitochondrial morphology (i.e., elongation) in the cell culture system as assessed by calculation of the FF and AR of individual mitochondrion in HCFs stained with Mitotracker Red (Yu et al., 2011; Figure 3d,e). Next, we injected AAV9‐hTCF21‐mt‐PKD‐DN or ‐Luc to adult rats. To avoid phenotypic changes during the plating and culturing process of primary CFs isolated from rat hearts (Zhang et al., 2011), mitochondrial morphology in CFs was assessed by TEM in fixed rat ventricular tissues. Cells located between CMs but not near/at the capillary structures were defined as CF‐like cells and cells located close to or possess capillary structures were more likely endothelial cells or smooth muscles (Bowers et al., 2012; Gherghiceanu & Popescu, 2012; Figure 3f). Similar to cultured HCFs, a significant increase in AR and mitochondrial perimeter (i.e., mitochondrial elongation) was observed in CF with AAV9‐hTCF21‐mt‐PKD‐DN compared to AAV9‐hTCF21‐Luc. No significant changes in mitochondrial morphology were observed in CMs (Figure 3g,h).

In summary, we demonstrated the potential of a simplified CF‐specific rat transgenic model using AAV9‐hTCF21 system. We successfully achieved a CF‐specific expression of transgene in adult rat hearts, but we also observed several off‐targeted infections in other organs, likely due to the nature of hTCF21 promoter activity outside of the heart. This model may allow us to develop fast and simple rat CF‐specific transgenic models of cardiovascular diseases in vivo.

## AUTHOR CONTRIBUTIONS

BN: Investigation and writing–review and editing. MWC: Investigation and writing–review and editing. BSJ: Conceptualization, methodology, validation, formal analysis, investigation, writing–review and editing, supervision, and funding acquisition. JOU: Conceptualization, methodology, validation, formal analysis, investigation, writing–original draft, writing–review and editing, supervision, and funding acquisition.

## FUNDING INFORMATION

No financial or otherwise, are declared by the author(s).

## CONFLICT OF INTEREST STATEMENT

No conflicts of interest, are declared by the author(s).

## ETHICS STATEMENT

All animal experiments were performed in accordance with the Guidelines on Animal Experimentation of the University of Minnesota (UMN) (Protocol#2105‐39060A). The study protocols were approved by UMN IACUC. The investigation confirmed the Guidelines for the Care and Use of Laboratory Animals published by the US NIH.

## Supporting information


Data S1:

## Data Availability

The data that support the findings of this study are available on request from the corresponding author. The data are not publicly available due to privacy or ethical restrictions.
