# Supplementary material for: Adeno‐associated virus‐based approach for genetic modification of cardiac fibroblasts in adult rat hearts
Source: Physiol Rep. 2024 Mar 27;12(6):e15989. doi: 10.14814/phy2.15989 (PMC10972676; doi:10.14814/phy2.15989)
Supplement: Supplementary file 1 — Data S1: [file PHY2-12-e15989-s001.docx]

**Supplementary Materials for**

**Adeno-associated virus-based approach for genetic modification of cardiac fibroblasts in rat hearts.**

Bridget Nieto, Michael W. Cypress, Bong Sook Jhun*, Jin O-Uchi*

**This PDF file includes:**

Supplementary Fig. S1

Supplementary Table 1 to 4

Supplementary References

**
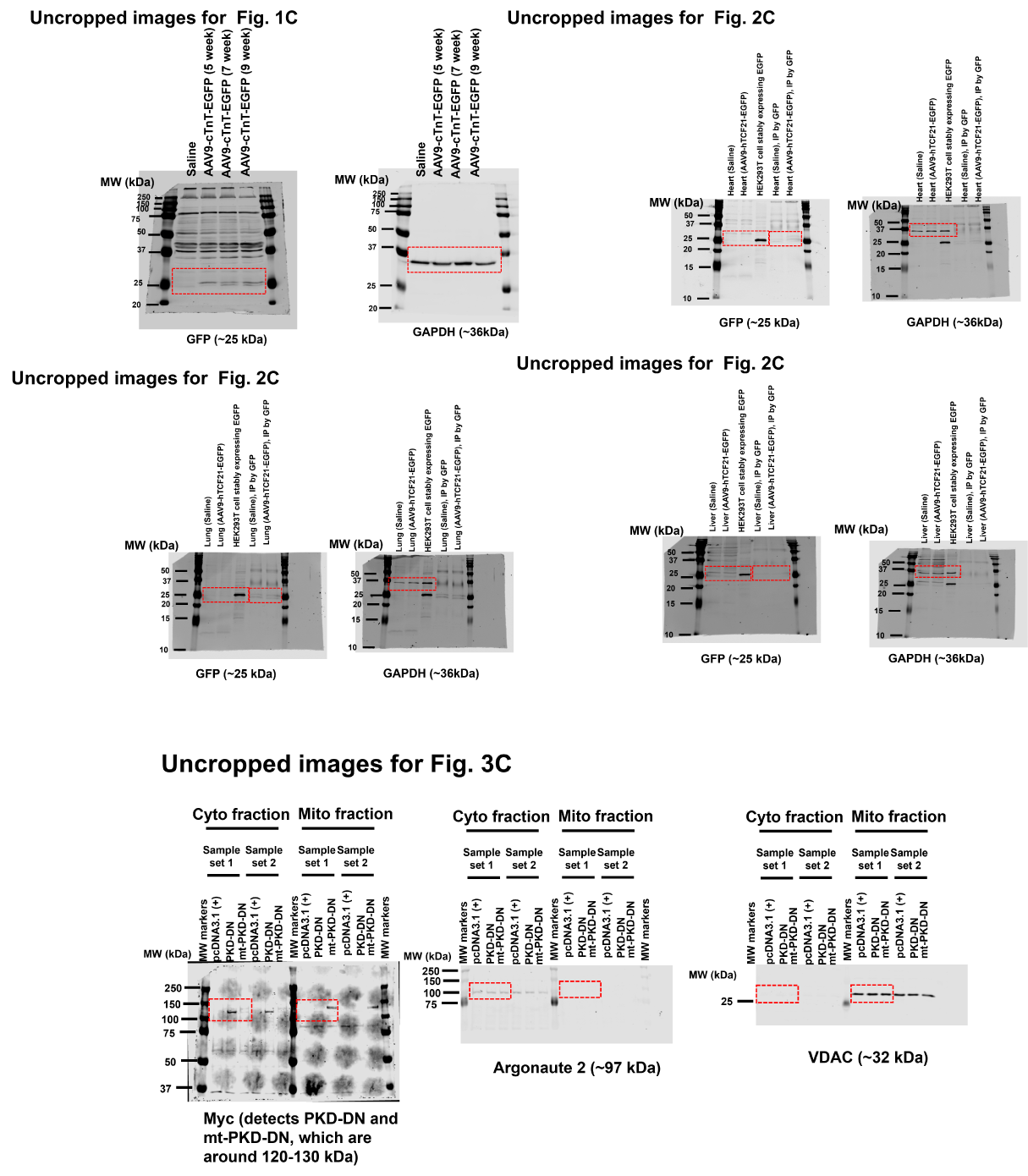
**

**Supplementary Fig. 1. Uncropped Western blotting images for Figures 1 to 3.**

Locations cropped for generating main figures are shown in dot squares.

**Supplementary Table 1. List of commercial primary antibodies used in this study.**

| **Targeted Protein** | **Type** | **Company** | **Catalog number** | **Immunogen** |
| --- | --- | --- | --- | --- |
| GFP | Mouse monoclonal | Sigma-Aldrich, St. Louis, MO and Roche Basel, Switzerland | 11814460001 | Recombinant *A. victoria* GFP |
| GFP | Rabbit monoclonal | Thermo Fisher Scientific, Waltham, MA | G10362 | Full-length GFP |
| vimentin | Mouse monoclonal | Sigma-Aldrich | V6630 | Pig eye lens vimentin |
| GAPDH | Mouse monoclonal | Cell Signaling Technology, Danvers, MA | 97166S | a synthetic peptide corresponding to residues near the amino terminus of human GAPDH protein |
| VDAC | Rabbit polyclonal | Cell Signaling Technology | 4866S | a synthetic peptide corresponding to the amino terminus of human VDAC-1 |
| Argonaute 2 (Argo2) | Rabbit monoclonal | Cell Signaling Technology | 2897S | A synthetic peptide corresponding to the amino terminus of human VDAC-1 |
| Myc | Mouse monoclonal | BioLegend, San Diego,CA | 626802 |  |

**Supplementary Table 2. List of plasmids used in this study.**

| **No** | **Inserted gene** | **Vector Backbone** | **Source/Provider** | **Company** | **Notes** | **Ref.** |
| --- | --- | --- | --- | --- | --- | --- |
| 1. | none | pEGFP-C1 |  | Clontech, Mountain View, CA |  |  |
| 2. | Mitochondrial matrix-targeted DsRed (mt-RFP) | pDsRed1-N1 | Dr. Yisang Yoon |  |  | (3) |
| 3. | Mitochondrial matrix-targeted GFP (mt-GFP) | pEGFP-N1 | Dr. Yisang Yoon |  |  | (3) |
| 4. | PKD1-K612W-GFP (PKD-DN-GFP) | pEGFP-N1 | Dr. Klaus Pfizenmaier, University of Stuttgart, Germany |  | PKD-DN can inhibit all three PKD isoforms (4) | (1) |
| 5. | HA/Myc-PKD1-K612W  (PKD-DN) | pcDNA3 | Dr. Alex Toker |  | Contains both HA and Myc tags. PKD1 has additional mutaions of Q66R, F139S and G877W | (1) |
| 6. | mt-HA/Myc-PKD1-K612W (mt-PKD-DN) | pcDNA3 |  | Vectorbuilder, Chicago, IL | Generated from No. 5 by adding amino acid 1-33 of human TOM20 at the N-terminus of PKD-DN |  |
| 7. | mt-PKD1-K612W-GFP (mt-PKD-DN-GFP) | pEGFP-N1 |  | Vectorbuilder | Generated from No. 4 by adding amino acid 1-33 of human TOM20 at the N-terminus of PKD1-DN-GFP |  |
| 8. | pAAV.cTNT.Luciferase | AAV2/9.cTnT.PI.EGFP.RBG |  | Addgene, Watertown MA | A gift from William Pu (Addgene plasmid # 69915; http://n2t.net/addgene:69915; RRID:Addgene_69915) | (2) |
| 9 | pENN.AAV.cTNT.PI.eGFP.WPRE.rBG | pAAV |  | Addgene | A gift from James M. Wilson (Addgene plasmid # 105543; http://n2t.net/addgene:105543; RRID:Addgene_105543) |  |
| 10. | Human TCF21 promoter fragment (1013bp) in pLightSwitch_Prom | pLightSwitch_ Promoter Reporter Vector |  | SwitchGear Genomics, Carlsbad, CA |  |  |
| 11. | pAAV[Exp]-{hTCF21 Promoter_1013bp}  >{HA/Myc  /hPRKD1*(K612W,Q66R,F139S,G877W)} | pAAV[Exp] |  | Vectorbuilder | Generated from No. 6 and 10 |  |
| 12. | pAAV[Exp]-{hTCF21 Promoter_1013bp}  >Luciferase:WPRE | pAAV[Exp] |  | Vectorbuilder | Generated from No. 10 |  |
| 13. | pAAV[Exp]-{hTCF21 Promoter_1013bp}  >EGFP | pAAV[Exp] |  | Vectorbuilder | Generated from No. 10 |  |
| 14. | pAV[Exp]-CMV>{hTOMM20[NM_014765.2]*(AA 1-  33)}:{HA/Myc/hPRKD1*(Mutation) | pAV[Exp] |  | Vectorbuilder | Generated from No. 6 |  |

**Supplementary Table 3. List of viruses used in this study.**

| **Name** | **Company** | **Notes** |
| --- | --- | --- |
| AAV9-cTnT-EGFP | Vectorbuilder | Generated from plasmid 9. Packaged by Vectorbuilder. |
| AAV9-cTnT-Luciferase | Vectorbuilder | Generated from plasmid 8. Packaged by Vectorbuilder. |
| AAV9-hTCF21-Luciferase | Vectorbuilder | Generated from plasmid 12. Packaged by Vectorbuilder. |
| AAV9-hTCF21-EGFP | Vectorbuilder | Generated from plasmid 13. Packaged by Vectorbuilder. |
| AAV9-hTCF21-mt-PKD-DN | Vectorbuilder | Generated from plasmid 11. Packaged by Vectorbuilder. |
| Adenovirus-FLAG | LifeSct, Rockville, MD |  |
| Adenovirus-mt-PKD-DN | Vectorbuilder. | Generated from plasmid 14. Packaged by Vectorbuilder. |

**Supplementary Table 4. List of key chemicals and reagents used in this study.**

| **Name** | **Company/Provider** | **Product #** | **Notes** |
| --- | --- | --- | --- |
| D-Luciferin Potassium Salt | Perkin Elmer, Waltham, MA | 122799 | *In vivo* bioluminescence imaging |
| Euthazol | Virbac, Westlake, Texas | EUTHASOL | Euthanasia |
| SuperScript IV VILO Master Mix | Thermo Fisher Scientific | 11756500 | Reverse transcription |
| Genious 2X SYBR Green Fast qPCR Mix (Low ROX Premixed) | ABclonal, Woburn, MA | RK21206 | Quantitative PCR |
| Applied Biosystems PowerUp SYBR Green Master Mix | Thermo Fisher Scientific | A25742 | Quantitative PCR |
| Fetal bovine serum | GIBCO, Grand Island, NY | 26140095 | Cell culture |
| 100 U/mL penicillin, and 100 µg/mL streptomycin | Genesee Scientific, El Cajon, CA | 25-512 | Cell culture |
| G418 | Corning, Corning, NY | 61-234-RK | Cell culture |
| Dulbecco's modified Eagle's medium (DMEM) | Cytiva, Marlborough, MA | SH30243.02 | Cell Culture |
| Human adult ventricular cardiac fibroblast growth medium | Cell Applications, San Diego, CA | 316-500 | Cell culture |
| H9c2 cells | ATCC, Manassas, VA | CRL-1446 | Cell culture |
| Human adult ventricular cardiac fibroblasts | Cell Applications, San Diego, CA | 306V-05a | Cell culture |
| HEK293T cells | Gift from Dr. Keigi Fujiwara, University Texas MD Anderson, Houston, TX | N/A | Cell Culture |
| Opti-MEM | Thermo Fisher Scientific | 51985034 | Cell transfection |
| FuGENE HD | Promega, Madison, WI | E2312 | Cell transfection |
| RNeasy Fibrous Tissue Mini Kit | Qiagen, Hilden, Germany | 74704 | mRNA isolation from rat tissues |
| Nitrocellulose membrane (0.45 μm pore size) | Genesee Scientific | 84-876 | Western Blotting |
| Nitrocellulose membrane (0.22 μm pore size) | Alkali Scientific, Fort Lauderdale, FL | XR642 | Western Blotting |
| Blocking buffer | Genesee Scientific | 20-314 | Western Blotting |
| Fluorescence-conjugated secondary antibodies | LI-COR Biotechnology | 925-32211 and 926-68020 | Western Blotting |
| Protein A/G Plus agarose beads | Santa Cruz Biotechnology, Dallas, TX | sc-2003 | Immunoprecipitation |
| MitoTracker Red | Thermo Fisher Scientific | M22425 | Live cell imaging |
| 16% Formaldehyde | Thermo Fisher Scientific | PI28908 | Immunohistochemistry |
| Goat serum | Cell Signaling Technology | 5425 | Immunohistochemistry |
| 2% glutaraldehyde in 0.1 M phosphate buffer | Electron Microscopy Sciences, Hatfield, PA | 16536-05 | Transmission electron microscopy |
| ProLong® Gold Antifade Reagent, With DAPI, Liquid | Cell Signaling Technology | 8961S | Immunohistochemistry |

**Supplementary References**

1.     **Jhun BS, O-Uchi J, Wang W, Ha CH, Zhao J, Kim JY, Wong C, Dirksen RT, Lopes CM and Jin ZG.** Adrenergic signaling controls RGK-dependent trafficking of cardiac voltage-gated L-type Ca2+ channels through PKD1. *Circ Res* 110: 59-70, 2012.

2.     **Lin Z, von Gise A, Zhou P, Gu F, Ma Q, Jiang J, Yau AL, Buck JN, Gouin KA, van Gorp PRR, Zhou B, Chen J, Seidman JG, Wang D and Pu WT.** Cardiac-specific YAP activation improves cardiac function and survival in an experimental murine MI model. *Circ Res* 115: 354-363, 2014.

3.     **Yoon Y, Krueger EW, Oswald BJ and McNiven MA.** The mitochondrial protein hFis1 regulates mitochondrial fission in mammalian cells through an interaction with the dynamin-like protein DLP1. *Mol Cell Biol* 23: 5409-5420, 2003.

4.     **Ziegler S, Eiseler T, Scholz RP, Beck A, Link G and Hausser A.** A novel protein kinase D phosphorylation site in the tumor suppressor Rab interactor 1 is critical for coordination of cell migration. *Mol Biol Cell* 22: 570-580, 2011.
